# Antenatal medication management for women with sickle‐cell disease: A systematic review

**DOI:** 10.1002/pmf2.70039

**Published:** 2025-06-05

**Authors:** Yaneve N. Fonge, Kelli D. Barbour, Sarah Boudova, Jen Sothornwit, Chetta Ngamjarus, Porjai Pattanittum, Apiwat Jongjakapun, Termtem Waidee, Nampet Jampathong, Pisake Lumbiganon, Beth L. Pineles

**Affiliations:** ^1^ Minnesota Perinatal Physicians Allina Health System Minneapolis Minnesota USA; ^2^ Department of Obstetrics and Gynecology Division of Maternal‐Fetal Medicine Baylor College of Medicine Texas USA; ^3^ Department of Obstetrics and Gynecology Division of Maternal‐Fetal Medicine Sidney Kimmel Medical College of Thomas Jefferson University Philadelphia Pennsylvania USA; ^4^ Department of Obstetrics and Gynecology Faculty of Medicine Khon Kaen University Khon Kaen Thailand; ^5^ Department of Epidemiology and Biostatistics Faculty of Public Health Khon Kaen University Khon Kaen Thailand; ^6^ Cochrane Thailand Khon Kaen University Khon Kaen Thailand; ^7^ Department of Obstetrics and Gynecology University of Pennsylvania Philadelphia Pennsylvania USA

**Keywords:** antenatal period, medications, perinatal period, pregnancy complications, sickle‐cell disease, systematic review, transfusion

## Abstract

**Background:**

Improvements in sickle‐cell disease (SCD) treatment have led to an increasing number of individuals surviving to reproductive age. However, pregnancy in women with SCD carries a heightened risk of maternal and fetal complications.

**Objective:**

To evaluate the effectiveness and safety of commonly used antenatal medications and transfusion strategies on maternal and neonatal outcomes in pregnant women with SCD.

**Methods:**

A systematic review was conducted using a pre‐registered protocol (PROSPERO: CRD42023484839). Comprehensive literature searches were performed in PubMed, Cochrane Central Register of Controlled Trials (CENTRAL), Scopus, Embase, and CINAHL from their inception to November 24, 2023. Eligible studies included randomized controlled trials (RCTs), cluster RCTs, quasi‐experimental studies, prospective and retrospective cohort studies, case‐control studies, interrupted time series, and controlled before‐and‐after studies. Included studies examined antenatal medication or transfusion strategies for pregnant women with SCD, including any genotype. Risk of bias was assessed using RoB 2 for RCTs and ROBINS‐I for non‐randomized studies (NRSIs). Certainty of RCT evidence was evaluated using GRADE criteria. Data were synthesized using narrative synthesis due to study heterogeneity.

**Results:**

A total of 23 studies were included, of which two were RCTs and 21 were NSRIs. The RCTs investigated prophylactic transfusion and iron supplementation. Prophylactic transfusion may reduce the risk of sickle‐cell crisis but the effect on other maternal or neonatal outcomes is unclear due to very low‐certainty evidence. The effect of iron supplementation on maternal and neonatal outcomes is also of very low certainty. The observational studies carried a high risk of bias preventing meta‐analysis and limiting the ability to draw conclusions.

**Conclusions:**

There is a limited body of evidence on antenatal medication and transfusion management strategies for pregnant women with SCD. High‐quality studies are needed to inform evidence‐based antenatal care for this population.

**Trial Registration:**

PROSPERO: CRD 42023484839

## 1 INTRODUCTION

### 1.1 Description of the condition: sickle cell disease

Sickle cell disease (SCD) is a group of autosomal recessive hemoglobinopathies caused by mutations in the β‐globin gene, resulting in the production of abnormal hemoglobin S (HbS) [1]. It is the most common inherited hemoglobin disorder globally, affecting approximately 7.74 million women^1^, with the highest prevalence in sub‐Saharan Africa, the Middle East/North Africa, India, Latin America, and Mediterranean countries [2]. However, migration and globalization have expanded the burden of SCD worldwide [3].

The clinical phenotype of SCD is characterized by chronic hemolytic anemia, intermittent vaso‐occlusive crises (VOCs), and progressive end‐organ damage due to recurrent ischemia and inflammation [3]. Disease severity varies by genotype, with homozygous HbSS being the most severe, while compound heterozygous forms (e.g., HbSC, HbSβ‐thalassemia) typically present with a milder course [4]. In contrast, most heterozygous individuals with sickle‐cell trait (HbAS) remain asymptomatic [3]. Advances in newborn screening, prophylactic interventions, and disease‐modifying therapies have improved survival [3, 5, 6]. From 1979 to 2016, the average life expectancy for women with SCD has increased from 36.9 years old to 55 years old [7, 8]. Thus, more women are with SCD are reaching reproductive age in good enough health to achieve pregnancy [9].

Pregnancy in women with SCD is associated with significant maternal and fetal morbidity due to the compounded effects of pregnancy‐related physiologic changes on an already compromised hematologic and vascular system [3, 6, 10]. Pregnant women with SCD are at increased risk of VOC, acute chest syndrome, osteonecrosis, hepatic necrosis, leg ulcers, and thromboembolic events [3]. Furthermore, vaso‐occlusion within the placenta can result in hypertensive disorders of pregnancy, fetal growth restriction, stillbirth, and other adverse perinatal outcomes [3, 6, 10]. Given these risks, optimizing antenatal management is essential for improving maternal and neonatal outcomes.

### 1.2 Description of the intervention and how it may work

The management of SCD in pregnancy requires a multifaceted approach that includes preventive care, symptom control, and treatment of disease‐related complications. Physiologic adaptations of pregnancy necessitate adjustments in therapeutic strategies to balance maternal and fetal safety. Several nutritional supplements, pharmacologic and transfusion‐based interventions have been proposed to mitigate complications, improve pregnancy outcomes, and reduce maternal morbidity. The specific interventions assessed in this review are listed in Box 1.

**BOX 1. Antenatal medication and transfusion interventions in SCD**

1. **Vitamin and mineral supplementation**
  - *Folic acid* is essential for DNA synthesis and cell division, making adequate levels crucial during pregnancy and in individuals with SCD to meet the increased metabolic demands of fetal development and red blood cell production. The appropriate dosing recommendations vary [11, 12, 13].
  - *Vitamin D* supports maternal and fetal bone health by regulating blood levels of calcium and phosphorus [14]. Patients with sickle cell are often deficient in vitamin D. It is uncertain if supplementation reduces obstetric, neonatal, or sickle cell specific complications.
  - *Iron supplementation* is routinely recommended in pregnancy, but its role in SCD remains debated due to the risk of iron overload from chronic hemolysis and transfusions, which can cause organ failure and mortality [15, 16, 17, 18, 19]. Outside of pregnancy, iron chelators can be used to treat iron overload, but iron chelators are not recommended during pregnancy due to concerns for fetal teratogenicity [20].
2. **Disease‐modifying therapy**
  - *Hydroxyurea* reduces SCD morbidity and mortality by increasing oxygen carrying capacity through increased production of fetal hemoglobin (HbF) decreasing vaso‐occlusive episodes and end‐organ damage [21]. In pregnancy, there is thought to be additional benefit through anti‐inflammatory properties that may reduce placental injury and insufficiency in pregnancies complicated by SCD [22, 23]. However, concerns about teratogenicity have limited its use during pregnancy [24, 25, 26].
3. **Blood transfusion therapy**
  - *Red blood cell transfusions* in people with SCD may be in the form of simple transfusions or exchange transfusions, either manual or automated. Simple transfusions consist of a transfusion of red blood cells only. Manual exchange involves manual phlebotomy followed by a transfusion. Automated exchange involves removal and replacement of red blood cells using an apheresis system. Transfusions are utilized to reduce HbS levels, improve oxygen delivery, and prevent sickle‐cell‐related complications. The role of prophylactic or serial transfusion in pregnancy is unknown but it is important to explore, given limited availability of disease‐modifying agents in pregnancy.
4. **Antiplatelet therapies**
  - *Low‐dose aspirin* outside of pregnancy has been shown to reduce the rate of sickle cell related complications [27]. It additionally has benefits in pregnancy in prevention of pre‐eclampsia, a condition to which SCD patients are particularly susceptible [28, 29].
5. **Thromboprophylaxis**
  - *Low‐molecular‐weight heparin (LMWH) and unfractionated heparin (UFH)* may be considered for thromboprophylaxis due to the hypercoagulable states of SCD and pregnancy. Pregnancy‐related thromboembolism risk is 11.7% in pregnant persons with SCD [30], but it is not clear when and if prophylactic anticoagulation should be used in pregnancy.
6. **Infection prevention and treatment**
  - *Antibiotics*: The splenic and renal sequelae of vaso‐occlusive crisis make patients with SCD particularly prone to infections [31]. This risk of infection is compounded by the immune tolerant state of pregnancy [32]. Antibiotic prophylaxis with penicillin has been studied and shown benefit in children and asplenic SCD patients [33, 34, 35], but it is not known if antibiotic prophylaxis should be used in pregnancy.
  - *Malaria prophylaxis*: Globally, malaria is the most common precipitating cause of crises and hospitalization [36]. In malaria‐endemic areas, chemoprophylaxis has been shown to reduce crises and mortality in SCD patients [36].
7. **Pain management**
  - *Opioids and non‐opioid analgesics* are used to manage acute and chronic SCD‐related pain in the general SCD population, but in pregnancy careful selection is required to minimize neonatal withdrawal and other adverse effects [37, 38, 39]. The ideal regimen for pain management in the inpatient and outpatient settings during pregnancy is unknown.

#### 1.2.1 Why it is important to do this review

With the increasing prevalence of SCD and improved survival into reproductive age, there is an urgent need for evidence‐based antenatal management strategies. While current guidelines [40, 41, 42, 43] recommend starting or stopping specific interventions, these are often based on low‐quality evidence or extrapolated from animal studies, non‐pregnant populations, or pregnant populations with related pathophysiology. The impact of these therapies on maternal and neonatal outcomes in pregnant people with SCD remains poorly defined, necessitating a systematic review to evaluate their efficacy and safety in pregnancy.

## 2 OBJECTIVES

The objective of this systematic review was to synthesize the existing evidence on antenatal medication and transfusion management in pregnant women with SCD. Specifically, we sought to evaluate the risks and benefits of vitamin and mineral supplementation, disease‐modifying therapy, blood transfusion therapy, antiplatelet therapies, thromboprophylaxis, antibiotics, malaria prophylaxis, and analgesics during pregnancy.

## 3 METHODS

### 3.1 Protocol

Prior to initiating this review, a protocol was developed detailing the search strategy, eligibility criteria, and methodological approach. The protocol was registered on PROSPERO International prospective registry of systematic reviews (CRD42023484839) on November 23, 2023. The review adhered to the Preferred Reporting Items for Systematic Reviews and Meta‐Analyses (PRISMA) guidelines throughout the process [44].

### 3.2 Eligibility and exclusion criteria

We searched for randomized controlled trials (RCTs), cluster RCTs, quasi‐experimental studies, prospective and retrospective cohort studies, case‐control studies, interrupted time series, and controlled before‐and‐after studies that included pregnant women with SCD, in any setting, regardless of genotype or disease severity. The studies had to examine antenatal medication or transfusion strategies outlined in Box 1. It was established a priori that the literature on SCD in pregnancy is limited; therefore, no specific comparisons were preselected. Any study evaluating the interventions of interest was considered for inclusion. No language restrictions were applied: non‐English publications were examined using Google Translate. We excluded conference abstracts forwhich associated full texts were not available. Duplicate reports were identified and excluded.

### 3.3 Information sources and search strategy

We searched for relevant studies in PubMed, Cochrane Central Register of Controlled Trials (CENTRAL), Scopus, Embase, and CINAHL from the inception of the respective database to November 24, 2023. The search terms used in each database are listed in the Supporting Information. References cited in relevant studies were manually searched for other potential articles. If necessary, study authors were contacted to obtain missing data.

### 3.4 Data collection and analysis

#### 3.4.1 Selection of studies

Study selection was performed in accordance with the Cochrane Handbook for Systematic Reviews of Interventions [45]. All records retrieved through electronic and manual searches were imported into Covidence (www.covidence.org) for screening. Duplicate records were removed. The remaining abstracts were independently reviewed by three reviewers (YF, BP, TW) to determine potential eligibility. The full texts of potentially eligible studies were then obtained and carefully assessed against the inclusion and exclusion criteria, documenting reasons for exclusion (YF and BP). Disagreements were resolved by discussion between two reviewers (YF and BP).

#### 3.4.2 Data extraction and management

We developed a data extraction form in Covidence, pilot‐tested it on two randomly selected manuscripts, and refined it accordingly. Four reviewers (YF, BP, KB, SB) independently extracted study characteristics and outcome data. When multiple analyses were presented, the intention‐to‐treat analysis was preferentially extracted. Extracted data were transferred to Review Manager 5 (RevMan 5) by an independent party, and YF verified the accuracy of the data against trial reports.

#### 3.4.3 Data synthesis

Data collected included publication type; funding source; noted conflicts of interest; study design; intervention of interest; sickle cell genotype; country; care delivery setting; number of participants; and baseline characteristics such as maternal age, gravidity, parity, race/ethnicity, weight, maternal baseline hemoglobin, maternal baseline reticulocyte count, and pregestational sequelae of SCD (splenectomy, retinopathy, thromboembolism, transfusion dependence, alloimmunization, pulmonary disease, avascular necrosis, painful crisis). Gestational age at delivery, mode of delivery, and infant birth weight were also collected. The primary outcomes assessed were maternal and neonatal morbidity and mortality. Secondary outcomes of interest pertained to health service utilization. A detailed breakdown of outcome measures is provided in Box 2.

**BOX 2. Outcome measures assessed in the review**

**Primary outcomes**

**Maternal outcomes**: Miscarriage, pyelonephritis, pneumonia, acute chest syndrome, thrombotic events, sickle cell crisis, pre‐eclampsia, eclampsia, maternal death, antenatal hospitalization, intensive care unit (ICU) admission, mode of birth, maternal well‐being, maternal satisfaction.

**Fetal/neonatal outcomes**: Congenital anomalies, stillbirth, neonatal death, perinatal death, neonatal ICU admission, gestational age at birth, preterm birth (< 32 weeks; < 37 weeks), birth weight, intrauterine growth restriction, low birth weight (< 2500 g), small‐for‐gestational‐age (< 10th centile for gestational age), Apgar score < 7 at 5 min, long‐term neurodevelopmental outcomes.

**Secondary outcomes**

**Health service utilization**: Antenatal hospital admissions and length of stay, emergency department visits, maternal ICU admission, length of postnatal hospitalization, neonatal hospitalization duration, costs of maternal and neonatal care.

#### 3.4.4 Measurement of treatment effect

We planned to pool data using a fixed‐effect model for low‐heterogeneity analyses and a random‐effects model where substantial heterogeneity was observed; however, no comparisons had more than one included study. We analyzed RCTs and NRSIs separately. For RCTS, the Mantel‐Haenszel method was used for dichotomous outcomes, and the inverse variance method was used for continuous outcomes. Statistical significance was set at *p* < 0.05. Analysis was performed in RevMan 5.4. For dichotomous outcomes, results were expressed as relative risk (RR) with 95% confidence intervals (CI). For continuous outcomes, we used mean difference (MD) with 95% CI. For NRSIs, we used effect direction tables and summarized results narratively.

#### 3.4.5 Assessment of heterogeneity

We planned to assess heterogeneity visually through forest plots and statistically using Chi‐squared tests and *I* ^2^ statistics. For *I* ^2^ > 50% or *p* < 0.10, heterogeneity would have been considered high. However, as noted previously, only one trial was included in each comparison.

#### 3.4.6 Assessment of risk of bias in included studies

We evaluated the risk of bias in the included studies in accordance with the Cochrane Handbook for Systematic Reviews of Interventions [45], using the Risk of Bias 2 (RoB 2) tool for RCTs and the Risk of Bias in Non‐Randomized Studies of Interventions (ROBINS‐I) tool for NRSIs. RoB 2 evaluates bias in five domains: randomization process, deviations from intended interventions, missing outcome data, outcome measurement, and selection of reported results [46]. ROBINS‐I assesses bias across pre‐intervention, intervention, and post‐intervention phases [47]. Responses to questions in these tools allows categorization of risk as low, some concerns, or high (RoB2); and low, moderate, serious, or critical (ROBINS‐I). Each article was assessed for risk of bias by two reviewers, selected from a team of four (YF, BP, KB, SB). Disagreements were resolved through discussion.

#### 3.4.7 Assessment of publication bias

We planned to assess publication bias using funnel plots where sufficient studies (≥ 10) were available [48]. However, given the small number of included studies, funnel plot asymmetry could not be formally evaluated.

#### 3.4.8 Summary of findings and certainty of evidence

For RCTs, a summary of findings (SoF) table was created in accordance with the Cochrane Handbook for Systematic Reviews of Interventions [45]. The certainty of evidence for each outcome was rated as “high,” “moderate,” “low,” or “very low” using the GRADE (Grading of Recommendations, Assessment, Development, and Evaluations) approach in GRADEpro GDT software [49].

#### 3.4.9 Sensitivity analysis

A sensitivity analysis was planned to exclude unpublished studies and studies with high or unclear risk of bias. However, sensitivity analysis could not be conducted because we only identified one RCT for each comparison and all NRSIs had a high risk of bias.

## 4 RESULTS

**FIGURE 1:**
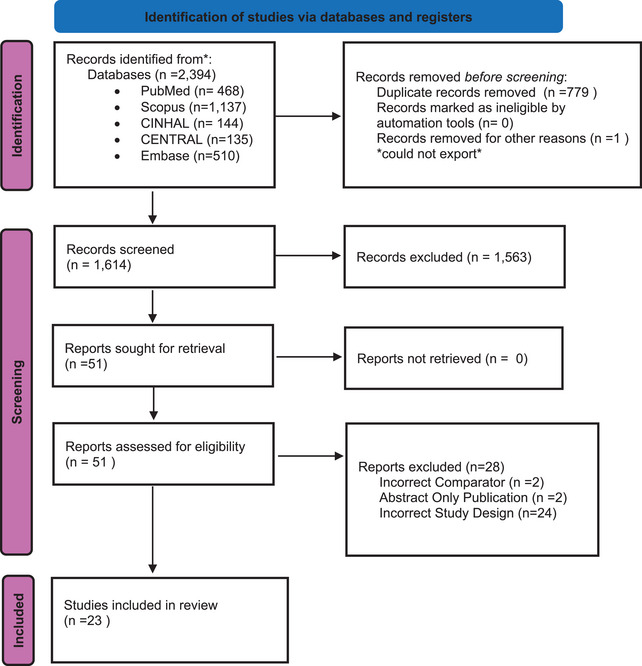
PRISMA. The total number of studies identified, excluded, and included in the final study is presented. Downward‐pointing arrows indicate next filter, and arrows pointing to the right indicate those who were excluded.

Figure 1 illustrates the project flowchart with included and excluded records and the reasons for their exclusions. In the end, 23 articles were included in the review consisting of two RCTs and 21 NRSIs. The results are presented below separating results from RCTs and NRSIs. No studies investigating secondary outcomes were identified.

### 4.1 Randomized studies

#### 4.1.1 Database search results

The searches identified two RCTs that are described in Table 1.

**TABLE 1:** Summary of characteristics of randomized controlled trials.

Ref (country)	Population	Number	Intervention	Comparator	Outcomes	Notes
	*1. Prophylactic red blood cell transfusion (simple or partial exchange) versus usual care*					
Koshy 1988 (United States of America)	Pregnant women with HbSS before 28 weeks’ gestation	Women:72 I = 36 C = 36 Newborns:76 I = 39 C = 37	Red blood cell transfusions at the beginning of pregnancy (commenced < 28 weeks) with the aim of maintaining hemoglobin between 6.21 and 6.83 mmol/L and reducing HbS to < 35% by simple transfusion or partial exchange transfusion. Transfusions received weekly for 3 weeks, or until goals reached.	Usual care (transfusion only for medical and obstetric indications)	Sickle‐cell crisis Splenic sequestration Acute chest syndrome Cesarean section Perinatal death Stillbirth Neonatal death Preterm birth Intrauterine growth restriction Apgar score < 7 at 5 min	Indications for blood transfusion in the control group were Hb < 3.72 mmol/L; hematocrit < 18%; reticulocyte count < 3% Study period: January 1979 to March 1986
	*2. Iron supplementation versus no iron supplementation*					
Akinyanju 1987 (Nigeria)	Pregnant women with SCD (11 HbSS and 3 HbSC)	Women: 14 I = 7 C = 7	Ferrous gluconate 300 g TID; folic acid 5 mg TID; proguanil hydrochloride 100 mg daily	Placebo; folic acid 5 mg TID; proguanil hydrochloride 100 mg daily	Sickle‐cell crisis Postnatal hemoglobin Birthweight	Proguanil hydrochloride is an anti‐malarial; Study period not stated; paper submitted in 1985

Abbreviations: C, control; I, intervention; Ref, reference; TID, three times daily.

The first trial, conducted in the United States between 1979 and 1986, compared prophylactic simple or partial exchange blood transfusion initiated in early pregnancy (< 28 weeks’ gestation) with usual care (indicated therapeutic blood transfusion) among pregnant women with HbSS (*n* = 72) [50]. The objective of the transfusion was to reduce sickle hemoglobin (HbS) to < 35%. However, the trial did not specify the number of women who received simple versus partial exchange transfusions.

The second trial, conducted in Nigeria, evaluated the effects of iron supplementation (ferrous gluconate 300 mg three times daily) administered from early pregnancy until immediately after birth compared with placebo among pregnant women with HbSS (*n* = 11) and HbSC (*n* = 3) [51]. Both groups received the same doses of folic acid and anti‐malarial prophylaxis. Although the study was published in 1987, the exact study period was not reported.

No RCTs were identified for other antenatal medications or transfusion relevant to the management of SCD in pregnancy.

#### 4.1.2 Synthesis of results

##### 4.1.2.1 Comparison 1: Prophylactic transfusion compared to usual care (indicated therapeutic transfusion)

###### 4.1.2.1.1 Risk of bias

According to the RoB 2 assessment, the trial by Koshy et al., which compared prophylactic simple or partial exchange blood transfusion with usual care, was rated as having some concerns for risk of bias. This was primarily due to some concerns related to the randomization process and the selection of reported results (Table 2).

**TABLE 2:** Summary of risk of bias for randomized controlled trial.

Study ID	Experimental	Comparator	Outcome	Weight	D1	D2	D3	D4	D5	Overall
Akinyanju 1987	Iron supplementation	Placebo	Sickle‐cell crisis	1	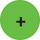	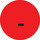	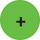	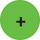	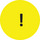	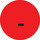
Akinyanju 1987	Iron supplementation	Placebo	Hemoglobin	1	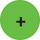	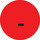	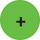	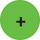	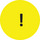	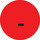
Akinyanju 1987	Iron supplementation	Placebo	Birthweight	1	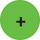	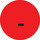	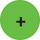	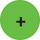	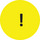	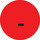
Koshy 1987	Prophylactic transfusion	Usual care	Sickle‐cell crisis	1	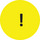	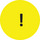	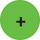	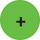	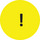	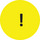
Koshy 1988	Prophylactic transfusion	Usual care	Sickle‐cell crisis	1	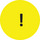	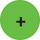	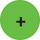	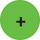	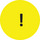	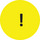
Koshy 1988	Prophylactic transfusion	Usual care	Splenic sequestration	1	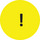	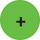	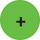	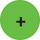	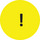	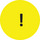
Koshy 1988	Prophylactic transfusion	Usual care	Acute chest syndrome	1	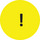	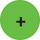	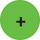	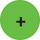	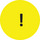	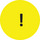
Koshy 1988	Prophylactic transfusion	Usual care	Cesarean section	1	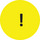	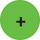	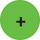	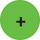	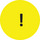	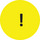
Koshy 1988	Prophylactic transfusion	Usual care	Gestational age at birth	1	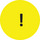	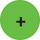	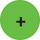	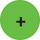	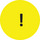	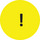
Koshy 1988	Prophylactic transfusion	Usual care	Perinatal death	1	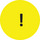	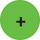	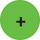	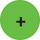	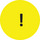	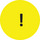
Koshy 1988	Prophylactic transfusion	Usual care	Stillbirth	1	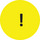	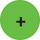	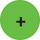	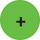	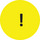	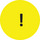
Koshy 1988	Prophylactic transfusion	Usual care	Neonatal death	1	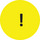	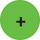	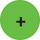	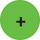	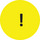	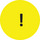

*Note*: The risk of bias for the included randomized controlled trials was assessed using Version 2 of the Cochrane Risk‐of‐Bias tool for randomized trials (RoB 2), the standard method for evaluating bias in Cochrane Reviews. RoB 2 examines specific domains related to trial design, conduct, and reporting, using predefined signaling questions to identify potential sources of bias. Based on these responses, the tool classifies the risk of bias for each domain as **“Low,” “High,”** or **“Some concerns.”** This tool was not applied to observational studies included in the review.

Abbreviation: D, domain.

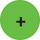
, Low risk; 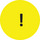, Some concerns; 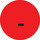, High risk; D1, Randomization process; D2, Deviations from the intended interventions; D3, Missing outcome data; D4, Measurement of the outcome; D5, Selection of the reported result.

###### 4.1.2.1.2 Baseline characteristics

The participants randomized to the prophylactic transfusion and usual care groups were well matched in terms of age, gravidity, parity, timing of the first clinic visit, gestational age at delivery, and smoking and alcohol use [50]. However, those in the prophylactic transfusion group on average had more hospital admissions (17.0 vs. 14.1, *p* < 0.05), more units of blood transfused (12 units vs. 3 units, *p* < 0.01), and delivered 2.3 weeks earlier than those receiving usual care (35.8 weeks vs. 38.1 weeks, *p* < 0.05) [50]. There was no significant difference in complications from transfusion including alloimmunization (transfusion: 10 (28) vs. usual care: 8 (22), *n* (%), *p* > 0.05) or transfusion reaction (transfusion: 6 (17) vs. usual care: 3 (8.3), *n* (%), *p* > 0.05) [50]. In most cases, insufficient data were provided to allow for re‐analysis, and *p*‐values were reported by the authors only as > 0.05 or < 0.05.

###### 4.1.2.1.3 Effects of interventions

Maternal and fetal/neonatal outcomes for prophylactic transfusion compared to usual care (indicated therapeutic transfusion) are summarized in Tables 3 and 4, respectively.

**TABLE 3:** Summary of findings: prophylactic transfusion compared to usual care—maternal outcomes for sickle‐cell disease.

Patient or population: Sickle‐cell disease Setting: United States of America Intervention: Prophylactic transfusion Comparison: Usual care—maternal outcomes						
	Anticipated absolute effects* (95% CI)					
Outcomes	Risk with usual care—maternal outcomes	Risk with prophylactic transfusion	Relative effect (95% CI)	No. of participants (studies)	Certainty of the evidence (GRADE)	Comments
Sickle‐cell crisis	500 per 1000	**140 per 1000** (60 to 335)	**RR 0.28** (0.12 to 0.67)	72 (1 RCT)	⨁⨁◯◯ (Low)^[Link]^, ^[Link]^	
Splenic sequestration	28 per 1000	**9 per 1000** (0 to 220)	**RR 0.33** (0.01 to 7.92)	72 (1 RCT)	⨁◯◯◯ (Very low)^[Link]^, ^[Link]^	
Acute chest syndrome	83 per 1000	**56 per 1000** (10 to 313)	**RR 0.67** (0.12 to 3.75)	72 (1 RCT)	⨁◯◯◯ (Very low)^[Link]^, ^[Link]^	
Caesarean section	278 per 1000	**222 per 1000** (100 to 497)	**RR 0.80** (0.36 to 1.79)	72 (1 RCT)	⨁◯◯◯ (Very low)^[Link]^, ^[Link]^	

^*^ **The risk in the intervention group** (and its 95% confidence interval) is based on the assumed risk in the comparison group and the **relative effect** of the intervention (and its 95% CI).

**CI**: confidence interval; **RR**: risk ratio; RCT: randomized controlled trial.

**GRADE Working Group grades of evidence** **High certainty**: we are very confident that the true effect lies close to that of the estimate of the effect. **Moderate certainty**: we are moderately confident in the effect estimate: the true effect is likely to be close to the estimate of the effect, but there is a possibility that it is substantially different. **Low certainty**: our confidence in the effect estimate is limited: the true effect may be substantially different from the estimate of the effect. **Very low certainty**: we have very little confidence in the effect estimate: the true effect is likely to be substantially different from the estimate of effect.

Explanations.

^a^Some concerns about study design.

^b^Estimate based on small sample size.

^c^Wide confidence interval including appreciable harm and appreciable benefit. Estimate based on small sample size and few events.

**TABLE 4:** Summary of findings: prophylactic transfusion compared to usual care—fetal/neonatal outcomes for sickle‐cell disease.

Patient or population: Sickle‐cell disease Setting: United States of America Intervention: Prophylactic transfusion Comparison: Usual care—infant outcomes						
	Anticipated absolute effects* (95% CI)					
Outcomes	Risk with usual care—infant outcomes	Risk with prophylactic transfusion	Relative effect (95% CI)	No. of participants (studies)	Certainty of the evidence (GRADE)	Comments
Perinatal death	54 per 1000	**154 per 1000** (33 to 715)	**RR 2.85** (0.61 to 13.22)	76 (1 RCT)	⨁◯◯◯ (Very low)^[Link]^, ^[Link]^	
Stillbirth	54 per 1000	**103 per 1000** (20 to 527)	**RR 1.90** (0.37 to 9.75)	76 (1 RCT)	⨁◯◯◯ (Very low)^[Link]^, ^[Link]^	
Neonatal death	0 per 1000	**0 per 1000** (0 to 0)	**RR 4.75** (0.24 to 95.76)	76 (1 RCT)	⨁◯◯◯ (Very low)^[Link]^, ^[Link]^	Rate in the prophylactic transfusion group was 5.6% (2/36), compared to 0% (0/36) in the usual care group
Preterm birth (< 37 weeks)	162 per 1000	**358 per 1000** (154 to 835)	**RR 2.21** (0.95 to 5.15)	76 (1 RCT)	⨁◯◯◯ (Very low)^[Link]^, ^[Link]^	
Intrauterine growth restriction	189 per 1000	**129 per 1000** (45 to 369)	**RR 0.68** (0.24 to 1.95)	76 (1 RCT)	⨁◯◯◯ (Very low)^[Link]^, ^[Link]^	
Apgar score < 7 at 5 min	81 per 1000	**154 per 1000** (41 to 571)	**RR 1.90** (0.51 to 7.04)	76 (1 RCT)	⨁◯◯◯ (Very low)^[Link]^, ^[Link]^	

^*^ **The risk in the intervention group** (and its 95% confidence interval) is based on the assumed risk in the comparison group and the **relative effect** of the intervention (and its 95% CI).

**CI**: confidence interval, RCT: randomized controlled trial; **RR**: risk ratio.

**GRADE Working Group grades of evidence** **High certainty**: we are very confident that the true effect lies close to that of the estimate of the effect. **Moderate certainty**: we are moderately confident in the effect estimate: the true effect is likely to be close to the estimate of the effect, but there is a possibility that it is substantially different. **Low certainty**: our confidence in the effect estimate is limited: the true effect may be substantially different from the estimate of the effect. **Very low certainty**: we have very little confidence in the effect estimate: the true effect is likely to be substantially different from the estimate of effect.

Explanations.

^a^Some concerns about study design.

^b^Wide confidence interval including appreciable harm and appreciable benefit. Estimate based on small sample size and few events.

^c^Wide confidence interval including appreciable harm and crossing the line of no effect. Estimate based on small sample size and few events.

Prophylactic transfusion may reduce the risk of sickle‐cell crisis compared to usual care (RR 0.28, 95% CI 0.12 to 0.67; 500/1000 prophylactic transfusion vs. 140/1000 usual care). However, the certainty of this estimate is low according to GRADE criteria, due to concerns about study design and imprecision. The effect of prophylactic transfusion on other sickle‐cell complications, including splenic sequestration (RR 0.33, 95% CI 0.01 to 7.92; 9/1000 prophylactic transfusion vs. 28/1000 usual care), acute chest syndrome (RR 0.67, 95% CI 0.12 to 3.75; 56/1000 prophylactic transfusion vs. 83/1000 usual care), or cesarean delivery (RR 0.80, 95% CI 0.36 to 1.79; 222/1000 prophylactic transfusion vs. 278/1000 usual care) is uncertain as these estimates are of very low certainty based on GRADE criteria. No other priority maternal outcomes were reported.

For fetal/neonatal outcomes, the effect of prophylactic transfusion compared to usual care on perinatal death (RR 2.85, 95% CI 0.61 to 13.22; 150/1000 prophylactic transfusion vs. 54/1000 usual care), stillbirth/fetal death (RR 1.90, 95% CI 0.37 to 9.75; 103/1000 prophylactic transfusion vs. 54/1000 usual care), neonatal death (RR 4.75, 95% CI 0.24 to 95.76; 0/1000 prophylactic transfusion vs. 0/1000 usual care), preterm birth (< 37 weeks) (RR 2.21, 95% CI 0.95 to 5.15; 358/1000 prophylactic transfusion vs. 162/1000 usual care), intrauterine growth restriction (RR 0.68, 95% CI 0.24 to 1.95; 129/1000 prophylactic transfusion vs. 189/1000 usual care), and Apgar score < 7 at 5 min (RR 1.90, 95% CI 0.51 to 7.04; 154/1000 prophylactic transfusion vs. 81/1000 usual care) is uncertain, again due to very low certainty evidence. No other priority newborn outcomes were reported.

##### 4.1.2.2 Comparison 2: Iron supplementation compared to placebo

###### 4.1.2.2.1 Risk of bias

The trial by Akinyanju et al., which evaluated iron supplementation versus placebo, was assessed as having high risk of bias. This was attributed to high risk of deviations from the intended intervention and some concerns about the measurement of the outcomes (Table 2).

###### 4.1.2.2.2 Baseline characteristics

No significant differences were found in terms of age (years) (iron 22.86 vs. placebo 24.42, *p* = 0.486) or maternal baseline hemoglobin (g/dl) (iron 8.17 vs. placebo 8.67, *p* = 0.478) between the iron supplementation and placebo groups.

###### 4.1.2.2.3 Effects of interventions

Maternal and fetal/neonatal outcomes for iron supplementation versus placebo are presented in Table 5. The effect of iron supplementation compared to placebo on the risk of sickle‐cell crisis (RR 0.50, 95% CI 0.06 to 4.33; 143/1000 iron vs. 286/1000 placebo) and postnatal maternal hemoglobin (MD 0.8 g/dL, 95% CI −1.03 to 2.63; 9.0 g/dL iron vs. 8.2 g/dL placebo) is uncertain due to concerns about study design and imprecision. No other priority maternal outcomes were reported. Regarding fetal/neonatal outcomes, the effect of iron supplementation compared to placebo on birthweight is also uncertain (MD 140 g, 95% CI −253.74 to 533.74; 2810 g iron vs. 2660 g placebo). No other priority newborn outcomes were reported.

**TABLE 5:** Summary of findings: iron supplementation compared to placebo for sickle‐cell disease.

Patient or population: Sickle‐cell disease Setting: Nigeria Intervention: Iron supplementation Comparison: Placebo						
	Anticipated absolute effects^*^ (95% CI)					
Outcomes	Risk with placebo	Risk with iron supplementation	Relative effect (95% CI)	No. of participants (studies)	Certainty of the evidence (GRADE)	Comments
Sickle‐cell crisis	286 per 1000	**143 per 1000** (17 to 1000)	**RR 0.50** (0.06 to 4.33)	14 (1 RCT)	⨁◯◯◯ (Very low)^[Link]^, ^[Link]^	
Postnatal maternal hemoglobin (g/dL)	The mean postnatal hemoglobin (g/dL) was 8.2	MD **0.8 higher** (1.03 lower to 2.63 higher)	–	14 (1 RCT)	⨁◯◯◯ (Very low)^[Link]^, ^[Link]^	
Birthweight (g)	The mean birthweight (g) was 2,660	MD **140 higher** (253.74 lower to 533.74 higher)	–	14 (1 RCT)	⨁◯◯◯ (Very low)^[Link]^, ^[Link]^	

^*^ **The risk in the intervention group** (and its 95% confidence interval) is based on the assumed risk in the comparison group and the **relative effect** of the intervention (and its 95% CI).

**CI**: confidence interval; **MD**: mean difference; RCT: randomized controlled trial; **RR**: risk ratio.

**GRADE Working Group grades of evidence** **High certainty**: we are very confident that the true effect lies close to that of the estimate of the effect. **Moderate certainty**: we are moderately confident in the effect estimate: the true effect is likely to be close to the estimate of the effect, but there is a possibility that it is substantially different. **Low certainty**: our confidence in the effect estimate is limited: the true effect may be substantially different from the estimate of the effect. **Very low certainty**: we have very little confidence in the effect estimate: the true effect is likely to be substantially different from the estimate of effect.

Explanations.

^a^Study design with high risk of bias.

^b^Wide confidence interval including appreciable harm and appreciable benefit. Estimate based on small sample size and few events.

^c^Wide confidence interval including appreciable harm and crossing the line of no effect. Estimate based on small sample size.

### 4.2 Non‐randomized studies

#### 4.2.1 Database search results

A total of 21 NRSIs also met inclusion criteria as summarized in Table S1 [52, 53, 54, 55, 56, 57, 58, 59, 60, 61, 62, 63, 64, 65, 66, 67, 68, 69, 70]. These included two retrospective studies with a historical comparison group [62, 70], two prospective studies with a concurrent comparison group [53, 57], 12 retrospective studies with a concurrent comparison group [52, 54, 56, 59, 60, 61, 63, 65, 66, 67, 68, 71], and three retrospective studies with a historical comparison group [55, 69, 72]. The interventions varied. Seven studies compared prophylactic red blood cell exchange with no prophylactic transfusion [52, 54, 56, 62, 63, 68, 70], while four compared prophylactic simple transfusion with no prophylactic transfusion [55, 65, 66, 71]. One study compared prophylactic simple transfusion at two hemoglobin cutoffs: < 10 g/dL versus < 6 g/dL [61]. Another study assessed prophylactic simple transfusion versus indicated simple transfusion plus oxygen therapy [72]. Additionally, one study of women exposed to hydroxyurea compared prophylactic simple transfusion with no prophylactic transfusion [61]. Hydroxyurea exposure was also explored in multiple contexts—two studies compared exposure at conception and during pregnancy to no exposure [57, 59]. One study compared both exposure at conception only and exposure during pregnancy only to the reference group of no hydroxyurea exposure [59].

One study investigated the effect of antimalarial chemoprophylaxis versus no antimalarial chemoprophylaxis [60].

No observational studies investigating other relevant antenatal interventions for management of SCD in pregnant women were identified.

#### 4.2.2 Risk of bias

Two studies were assessed to be at critical risk of bias [53, 67] and the remainder were judged to be at serious risk of bias due to confounding and insufficient information on the selection of reported results, as no study protocols were available (Table S1). This level of bias, combined with heterogeneity in study design, small sample sizes, uneven group distributions, and the lack of data adjusted for confounding factors, precluded pooling and meta‐analysis. Any findings from these studies should be interpreted with caution, as they may not establish a true causal relationship between the interventions and outcomes.

#### 4.2.3 Synthesis of results from non‐randomized studies

The results from the NRSIs are summarized in Table S2. In the seven studies comparing prophylactic red blood cell exchange with no prophylactic transfusion, there was a suggestion of reductions in odds of maternal death [52, 68], SCD complications [63], intrauterine growth restriction [56], preterm birth [63, 68, 70], perinatal death [63, 70], stillbirth [68], neonatal death [68], and low birthweight/small for gestational age [63, 70]. Three studies comparing prophylactic simple transfusion with no prophylactic transfusion suggested reductions in SCD complications [65], preterm birth [65], and perinatal death [55]. One study comparing prophylactic simple transfusion thresholds of hemoglobin < 10 g/dL versus < 6 g/dL reported increased odds of SCD complications when the threshold for transfusion is set at hemoglobin < 10 g/dL [69] One study comparing prophylactic simple transfusion with no prophylactic transfusion in women exposed to hydroxyurea at conception suggested an reduction in SCD complications [61]. One study found that hydroxyurea at conception and during pregnancy compared with no exposure was associated with an increase in odds of miscarriage and low birthweight/small for gestational age. Antimalarial chemoprophylaxis was associated with a reduction in low birthweight compared to no prophylaxis [60].

## 5 DISCUSSION

### 5.1 Principal findings

This systematic review identified a limited body of evidence on antenatal management strategies for pregnant women with SCD. The review aimed to evaluate the effects of multiple antenatal medication and transfusion strategies on maternal and fetal/neonatal outcomes. However, the included studies were limited to interventions involving transfusion, iron supplementation, hydroxyurea, and antimalarials. Notably, no studies met the inclusion criteria for other relevant interventions, including pain management, folic acid, vitamin D, aspirin, anticoagulation, or antibiotics, indicating substantial knowledge gaps. Additionally, no eligible studies were found that reported on our secondary outcomes of interest, healthcare utilization outcomes.

Two RCTs were identified: one investigating prophylactic transfusion [50] and the other iron supplementation [51]. Prophylactic transfusion may reduce the risk of sickle‐cell crisis; however, the effect on other maternal or neonatal outcomes is unclear. The certainty of this evidence was rated as very low to low according to GRADE criteria, due to concerns about study design and imprecision. Similarly, the effect of iron supplementation during the antenatal period on maternal and neonatal outcomes was uncertain due to small sample size (*n* = 14), study design, and imprecision.

Most available studies were NRSIs and carried a serious risk of bias due to confounding, selection bias, and inconsistent outcome measures, which precluded meta‐analysis. The evidence base was further constrained by heterogeneity in study designs and the absence of standardized outcome measures, which hindered the ability to draw definitive conclusions. The applicability of the NRSI findings to clinical practice is therefore limited.

### 5.2 Results in the context of what is known

There are no systematic reviews on antenatal medication management of SCD during pregnancy, but several international guidelines have summarized the literature. Our findings are in line with existing clinical guidelines, which predominantly rely on expert consensus and indirect evidence due to the scarcity of high‐quality evidence on antenatal management strategies for SCD during pregnancy [40, 41, 42, 43]. In the past 14 years, guidance on antenatal medication and transfusion strategies has been published by three groups: the Delphi Consensus on SCD [42], the Society for Maternal‐Fetal Medicine (SMFM) [43], and the British Society of Haematology (BSH) [41]. These groups generally agree on the utility of vitamin and mineral supplementation (including folic acid, vitamin D, and iron), and low‐dose aspirin for preeclampsia prevention. However, they vary in their recommendations for disease‐modifying agents, transfusion strategies, and anticoagulation, highlighting areas in which further research should be prioritized.

The Delphi consensus summarized expert survey responses on clinical scenarios and statements developed from data obtained through a systematic literature review on SCD and related complications in pregnancy [42]. Panelists completed digital questionnaires, with the first round featuring open‐ended questions across seven topics, and the second round using close‐ended questions formulated from the initial responses, supplemented by relevant medical literature [42]. Consensus was defined as ≥75% agreement among panelists [42]. The panelists included 12 international multidisciplinary experts from Belgium, France, India, Turkey, the United Kingdom, and the United States [42]. The panel achieved consensus on several key aspects of antenatal management, recommending monthly multidisciplinary antenatal follow‐up, prophylactic low‐dose aspirin for pre‐eclampsia prevention, and the use of prophylactic transfusion therapy in certain high‐risk scenarios [42]. However, no consensus was reached on critical areas such as the continuation of hydroxyurea, target hemoglobin levels, routine use of prophylactic transfusions, and the role of transfusions for preventing fetal complications [42]. Additionally, there was no agreement on the use of antenatal anticoagulation outside of usual care scenarios, such as women with a history of VTE, genetic predispositions, or risk factors like immobility or hospitalization [42]. These discrepancies in provider opinions underscore the challenges in clinical decision‐making due to the limited evidence base.

The SMFM Practice Statement recommends that use of prophylactic transfusions be individualized for high‐risk patients with SCD, guided by a hematologist and maternal‐fetal medicine specialist through shared decision‐making with the patient [43]. It also advises an individualized shared decision‐making approach regarding hydroxyurea use during pregnancy, considering the timing of use, disease severity, and alloimmunization status [43]. However, the recommendations are graded as 2B and 1C, reflecting low certainty due to the reliance on expert consensus rather than robust evidence [43]. Additionally, SMFM recommends considering prophylactic anticoagulation to reduce the risk of VTE for pregnant patients with SCD who are admitted to the hospital with anticipated decreased mobility or other risk factors, as well as those who would require anticoagulation independently of their SCD [43].

BSH, on the other hand, advises discontinuing hydroxyurea when attempting to conceive unless the woman is at high risk of severe complications and blood transfusion is not feasible [41]. Unlike the SMFM practice statement [43] and the Delphi Consensus on SCD [42], BSH guidelines recommend prophylactic transfusion only for women with severe disease, multifetal pregnancies, or those receiving long‐term transfusions for stroke prevention [41]. BSH agrees with Delphi and SMFM in recommending prophylactic anticoagulation for women with SCD who have risk factors such as immobilization or hospitalization [41]. They go a step further, however, advising starting prophylactic low molecular weight heparin (LMWH) from 28 weeks of pregnancy and, for women with additional risk factors, beginning at the start of pregnancy [41]. Like other guidelines, the strength of their recommendations remains weak (GRADE 2C), reflecting the reliance on low‐certainty evidence derived from expert opinion and indirect data [41].

Two other guidelines on SCD in the general population have also addressed the unique challenges associated with pregnancy in this population [73, 74]. Consistent with SMFM's practice statement [43], the Delphi Consensus on SCD [42], and BSH's guidelines, they conclude that there is insufficient evidence to recommend universal prophylactic transfusion [73, 74]. Additionally, they highlight concerns about the lack of evidence to guide recommendations on transfusion and anesthetic care in pregnant women, particularly during birth and pain crises [73, 74].

### 5.3 Implications for clinical practice

This review identifies substantial gaps in evidence regarding the safety and efficacy of hydroxyurea, anticoagulation, antiplatelet medications, pain management, vitamin D, folate, iron supplementation, malaria prophylaxis, antibiotic prophylaxis, and transfusion strategies in pregnant women with SCD. The lack of robust data presents significant clinical challenges, as healthcare providers are left with limited guidance when making treatment decisions.

This is particularly relevant for interventions like iron supplementation, where practice is complicated by the unique physiology of SCD. While iron is routinely recommended in pregnancy, its use in SCD remains debated due to the risk of iron overload from chronic hemolysis and transfusions. Without clear, evidence‐based guidance, clinicians must rely on individualized assessment, including iron status evaluation, to balance potential benefits and risks.

A key area of concern involves disease‐modifying agents such as hydroxyurea, which are frequently discontinued during pregnancy due to concerns about teratogenicity. These concerns largely stem from animal studies conducted at doses significantly higher than those prescribed to humans for SCD [75]. Moreover, existing studies linking adverse outcomes to hydroxyurea exposure failed to stratify results by the duration of exposure. Poor outcomes in these studies may be more reflective of suboptimal disease control rather than a direct effect of the medication as most people in the intervention group stopped the intervention in the first trimester [57]. Pregnancy outcomes in chronic diseases are better when the disease is well under control, but in the absence of high‐quality evidence, healthcare providers face uncertainty and may adopt a more cautious approach, discontinuing medications that could potentially optimize maternal and fetal outcomes.

Similarly, the risk of VTE in pregnant women with SCD is now understood to be higher than previously estimated, with rates as high as 11.3% for VTE and 5.2% for arterial thrombosis, particularly among those with poorly controlled disease [76]. Despite this elevated risk, the absence of rigorous data on the safety and efficacy of anticoagulation in pregnancy complicates decision‐making around prophylaxis. This is especially significant given that the VTE risk in SCD exceeds that of other high‐risk thrombophilias for which anticoagulation is routinely recommended during pregnancy [77]. Without definitive guidance, providers must navigate complex risk‐benefit analyses, potentially leading to variability in care practices.

This variability can contribute to disparities in access to care and resource allocation. In low‐resource settings, access to safe blood transfusions and comprehensive antenatal care remains limited [42]. Conversely, in high‐income countries where SCD is considered a rare disease, affected individuals frequently encounter socioeconomic barriers to healthcare access, particularly in systems lacking universal coverage [42]. Without high‐quality evidence to define best practices, inconsistent management approaches can emerge. Standardized toolkits and clinical protocols have proven effective in reducing maternal morbidity and mortality by providing evidence‐based guidance and minimizing variability in care [78]. However, the lack of high‐quality evidence for SCD‐specific antenatal management hampers the development of such standardized approaches. Robust, adequately powered RCTs are urgently needed to establish evidence‐based clinical guidelines, promote equitable access to care, and optimize antenatal management for pregnant women with SCD.

### 5.4 Evidence published since the review

Since the completion of this systematic review, a randomized study of prophylactic exchange transfusion versus standard care (no prophylactic red blood cell exchange) was published [79]. This small feasibility trial included 34 pregnant women with SCD across seven hospitals in the United Kingdom. No conclusive indications of effect were found due to wide confidence intervals and a small sample size, but a trend toward a lower incidence of VOC and preterm delivery, and improved birthweight were noted with prophylactic exchange transfusion [79]. Additionally, an ongoing trial in Lagos, Nigeria is investigating the role of low dose aspirin in preventing preeclampsia and fetal growth restriction in pregnant women with SCD [80]. The trial is expected to report on other priority outcomes, including VTE, maternal death, preterm delivery, perinatal death, number of crises, need for blood transfusion, and complications such as infections and placental abruption [80].

### 5.5 Strengths and limitations

#### 5.5.1 Strengths

This systematic review followed rigorous Cochrane methodology, including a pre‐registered protocol, a comprehensive search strategy across multiple databases, and a systematic risk of bias assessment using validated tools (RoB 2 and ROBINS‐I). Additionally, a broad range of study designs was included to capture the limited literature on SCD in pregnancy.

#### 5.5.2 Limitations

We acknowledge several limitations. Most included studies were observational, with high risk of bias. Additionally, the scarcity of high‐quality RCTs limited the ability to make evidence‐based comparisons, and no studies included pregnancy‐specific data for certain interventions. The variability in study designs and outcome reporting prevented meta‐analysis, further constraining the synthesis of evidence.

Many studies were outdated, limiting the evaluation of newer management options, including disease‐modifying agents like crizanlizumab and L‐glutamine. Two studies also had missing data that could not be obtained from the authors, impacting the completeness of the findings [54, 67]. Moreover, the geographic and temporal context of the included studies may limit the generalizability of results to current clinical practice. Finally, the inclusion of all SCD phenotypes, while necessary due to small sample sizes, limits the ability to draw phenotype‐specific conclusions—an important constraint given that different SCD genotypes can have distinct clinical courses and risks during pregnancy.

### 5.6 Conclusion

Overall, the completeness and applicability of the included data is limited. The evidence is insufficient to draw conclusions on appropriate antenatal medication and transfusion management in SCD. The lack of high‐quality RCTs and consistent outcome measures underscores the urgent need for well‐designed, adequately powered trials to evaluate the safety and efficacy of antenatal interventions in SCD.

## CONFLICT OF INTEREST STATEMENT

The authors declare no conflicts of interest.

## Supporting information



Supporting Information

## ENDNOTE

1 In this text, the word woman refers to all individuals capable of pregnancy.
